# Add-On Complementary Medicine in Cancer Care: Evidence in Literature and Experiences of Integration

**DOI:** 10.3390/medicines4010005

**Published:** 2017-01-24

**Authors:** Elio Rossi, Mariella Di Stefano, Fabio Firenzuoli, Maria Valeria Monechi, Sonia Baccetti

**Affiliations:** Tuscan Network of Integrative Medicine (TNIM), Regione Toscana, Assessorato al Diritto alla Salute, al Welfare e all’Integrazione socio-sanitaria, Direzione Diritti di cittadinanza e coesione sociale, Via Taddeo Alderotti 26/N, Firenze 50139, Italy; m.distefano@mednat.it (M.D.S.); fabio.firenzuoli@unifi.it (F.F.); valeriamonechi@gmail.com (M.V.M.); sonia.baccetti@uslcentro.toscana.it (S.B.)

**Keywords:** cancer care, complementary medicines (CM), evidence in literature, integration in public health service

## Abstract

**Background**: According to the literature an increasing number of cancer patients demand for complementary therapies during their disease. Research has demonstrated that some of these therapies are effective and safe as adjunctive treatments in specific symptoms of these patients. **Methods**: The aims of the paper are to review the main and recent papers of international literature on the effectiveness of complementary medicine (CM) therapies on side effects of anti-cancer protocols and improvement in the quality of life of oncological patients, and to describe the integration of evidence-based acupuncture, herbal medicine and homeopathy treatments in Public Cancer Network of the region of Tuscany. **Results**: After the review of literature and the approval of a Regional Resolution, some CM will be introduced in Cancer Departments in Tuscany to additionally treat cancer-related symptoms and side effects of conventional cancer therapy: acupuncture for nausea and post-chemotherapy and post-surgery vomiting, pain, hot flashes of iatrogenic menopause, xerostomia; homeopathy for hot flashes of iatrogenic menopause and the side effects of radiotherapy; herbal medicine for cancer-related fatigue, nausea and vomiting, pain, mucositis, anxiety, and depression. **Conclusions**: The integration of evidence-based complementary treatments allows for an effective response to the demand coming from cancer patients and combines safety and equity of access in public health systems.

## 1. Introduction

Numerous studies report that in Europe one cancer patient out of three turns to add-on CM, also called Complementary and Alternative Medicine (CAM) or Integrative Medicine (IM), in combination with conventional therapies.

According to Molassiotis et al. [1] the use of complementary therapies in Europe varies from 15% to 73% and the most widely used treatments are homeopathy, herbal medicine and spiritual therapies. The French study of Simon et al. [2] reported in 2007 that nearly 28% of 244 cancer patients from 2 public hospitals used one or several CAM, especially homeopathy (60%). A study carried out in two Tuscan hospitals on patients receiving chemotherapy [3] reported an incidence of 17% (herbal medicine 52%, homeopathy 30%, acupuncture 13%) in the use of non-conventional medicine.

According to the study on the use of CM in Italian cancer patients in six cancer departments and on the perception of the benefits of these therapies [4], 37.9% of patients use one or more types of CM: diet and food supplements (27.5%), herbal medicine (10.8%), homeopathy (6.4%) and body-mind therapies (5.5%); a high percentage of patients (66.3%) also inform the physicians of this choice and the benefits they experience (89.6%).

These therapies are also used in the field of paediatrics. According to an Italian study on the use of CM in children hospitalized at the Institute of Tumors in Milan [5], 12.4% had used at least one of these therapies, especially to reduce the side effects of conventional treatments.

In the last decade, in order to meet the requests of cancer patients, departments of integrative oncology have been set up in the main US hospitals (Dana-Farber Cancer Institute in Boston, Memorial Sloan-Kettering Cancer Center in New York and MD Anderson Cancer Center in Houston, among others). A survey conducted in 2014 by Rossi et al. estimated that about 20% of the European oncological structures also deliver complementary treatments [6].

## 2. Evidence of Effectiveness and Use in Cancer Care: Acupuncture and Traditional Chinese Medicine

A review of the literature on the subject of acupuncture and traditional Chinese medicine (TCM) was carried out at the Centre of TCM “Fior di Prugna” of the Tuscany Health Unit Center from 2003 to date. The scientific papers published were evaluated by taking into account the grading system of the Society for Integrative Oncology (SIO) [7], which defines the level of effectiveness of the treatments and the strength of the recommendations based on the level of scientific evidence (A, B, C) and on the benefits/risks/costs ratio. The grade of recommendation (1A, 1B, 1C, 2A) derives from the sum of the two criteria.

Since this grading was first published in 2009, the classification has been updated by the authors taking into account the most recent works and using the same basic criteria.

The major evidences of effectiveness are: National Cancer Institute 2013 [8], Filshie and Hester, 2006 British Guidelines [9], Society of Integrative Oncology (SIO) 2009 Guidelines [6], and National Comprehensive Cancer Network (NCCN) Clinical Practice Guidelines in Oncology^TM^ Palliative Care 2010, 2014 [10] all consider acupuncture an appropriate treatment for nausea and for post-chemotherapy vomiting.

Some studies [11,12] involved women with breast cancer who underwent chemotherapy and antiemetic therapy combined with acupuncture in various forms; other studies concerned lung or other types of cancers. There is much evidence for CM effectively treating nausea and post-surgical and post-radiotherapy vomiting [13]. The SIO 2009 grading system awarded 1A (strong recommendation, high quality evidence).

Acupuncture treatment (alone or added to painkillers) has a 1A grade also for treating cancer pain. Some studies show the effectiveness of acupuncture in the post-thoracotomy lung cancer pain, in joint pain from aromatase inhibitors [14], in pancreatic and in neck cancer pain.

Numerous systematic reviews claim that “acupuncture can be an important adjunctive treatment in patients with cancer pain, especially in non-responders, despite the methodological problems of the studies.”

The same conclusion is reached by the US National Cancer Institute, 2013 [8]. For this reason, the English Guidelines of 2006 [9], the SIO Guidelines of 2009 [7] and those of the NCCN in 2013 [10] recommend the use of acupuncture or acupressure, together with pharmacological interventions, in a multimodal approach to pain management. These integrated interventions, according to the NCCN, may be particularly important in vulnerable populations (frail patients, elderly patients, children). The guidelines for lung cancer conclude that in pain and peripheral neuropathy, acupuncture is suggested as an adjunctive treatment in patients with inadequate control of symptoms.

The use of acupuncture is also very common in the treatment of post-surgical, post-chemotherapy or ongoing hormonal therapy for hot flashes in women with breast and ovary cancer and, to a lesser extent, of patients with prostate cancer. The aforementioned Filshie and Ester Guidelines [9] indicate that acupuncture should be used in breast, prostate and other cancers. The SIO Guidelines [7] state that acupuncture does not seem to be more effective than sham acupuncture for the treatment of hot flashes, but that this treatment should be considered in patients with severe symptoms and those who are not responding to treatment. The degree of recommendation is 1B (strong recommendation, moderate quality evidence).

The Cochrane review of 2013 [15] evaluated the effects of acupuncture on hot flashes and on the quality of life in women in physiological and iatrogenic menopause. A total of 16 randomised controlled trials (RCTs) was taken into account, with 1155 women in perimenopause/menopause. The authors considered the studies separately, according to the control group used, and highlighted that in the comparison between acupuncture and sham acupuncture the differences were not significant in terms of the frequency of hot flashes, but that the hot flashes were significantly less acute in the acupuncture group, although the effects of the acupuncture treatment were small.

With regard to hormone therapy, the acupuncture groups presented a greater frequency and equal intensity of hot flashes. On the contrary, there were positive effects in the case of a wait-list or no-intervention control group. However, many authors believe that sham acupuncture is not a true placebo, since it is able to determine effects similar to those of acupuncture and that, when it is chosen to be used for the control group, it can cause an underestimation of the effectiveness of “real” acupuncture [15,16,17,18,19]. In conclusion, treatment with acupuncture may be promising for menopausal symptoms, especially for women who cannot take hormone replacement therapy because of cancer risks or past oncological diseases.

In a review and meta-analysis that examined 12 RCTs (672 women), Chen et al. [20] found a reduction in the number of hot flashes after treatment and at follow-up, compared to the controls in women with breast cancer. They also observed an improvement in the quality of life, although the low quality and the number of studies should lead to a cautious interpretation of these results. In conclusion, treatment with acupuncture may be considered a promising therapeutic technique; it is recommended for women who cannot take hormone therapy because of cancer risks or their present or past neoplastic pathology. It is also a preferable treatment for cases when the patient refuses or has already been treated with hormone therapy but has hot flashes, psychological problems and insomnia.

The treatment of xerostomia, caused by radiation therapy, especially in patients with head and neck tumors, was effective with SIO 1B grading (strong recommendation, evidence of moderate quality); an increase in salivation and an improved quality of life were also highlighted. Filshie and Ester Guidelines [9] indicate that xerostomia can be treated with acupuncture in patients who do not respond to conventional treatments. In a Cochrane review (2013) on non-pharmacological interventions for the treatment of xerostomia, Furness et al. [21] included five RCTs on the acupuncture (153 participants) of patients with xerostomia caused by radiation therapy (for the majority) or by Sjogren’s Syndrome. With regard to the amount of stimulated whole saliva (SWS), two studies showed a benefit in favor of acupuncture (*p* = 0.002, I^2^ = 1%), which persisted at follow-up at 12 months (SWS, *p* = 0.004, I^2^ = 0%). The authors conclude that there is some evidence that acupuncture produces a small increase in the production of saliva in patients with xerostomia caused by radiation therapy, persistent at one year after treatment, although the quality of the studies is low. Additionally, the adverse effects are short-term and mild.

The treatment of anxiety, depression, and insomnia (graded 2B), and of fatigue, neuropathy, lymphoedema, and leucopenia (graded 2C) are found to have lower gradings, although they are present in fewer studies and of lower quality.

The review conducted by Chien et al. [22] in 2013 on insomnia considered a number of studies evaluating the effectiveness of somatic acupuncture and of auriculotherapy in cancer patients with positive results for both techniques. According to the authors, the studies on insomnia are difficult to interpret owing to confounding factors that are often present, but the data available show the sedative and hypnotic effects of acupuncture when it is used in the treatment of insomnia and nervousness.

The review by Haddad and Palesh [23] on acupuncture for cancer-related psychological symptoms assessed four studies on sleep disorders with positive results. However, the important methodological problems (no randomization for some, low number of participants, undefined protocol according to the Standards for Reporting Interventions in Clinical Trials of Acupuncture-Stricta-criteria) require further well-designed studies.

As for anxiety and depression, the review by Chandwani et al. [24] analyzes the stress related to CAM-treated cancer. Among the techniques considered (yoga, meditation, tai chi chuan, etc.), the authors cite five studies that use acupuncture, and conclude that it can reduce anxiety, fatigue and stress associated with advanced cancer.

Garcia et al. in their review of 2013 [11] report that, although acupuncture is often used to manage anxiety and stress, relatively few studies have evaluated its use for these symptoms in cancer patients. The authors analyze six trials, five of which have been successful, but they were not “blinded” and therefore were evaluated as having a high risk of bias (ROB).

In 2014, Haddad and Palesh [23], in their review of 12 papers on acupuncture used for psychological symptoms related to cancer, report six studies for depressive symptoms and four for anxiety symptoms. These show promising positive effects for depressive symptoms, but the methodological problems (no randomization for some, low number of participants, undefined protocol according to the Stricta criteria) make it necessary to perform well-designed studies to demonstrate the effectiveness of acupuncture which, however, remains a treatment with fewer side effects than pharmacological therapies.

Few high-quality RCTs have been published on cancer-related fatigue (graded 2C) to evaluate the effectiveness of acupuncture and moxibustion which, however, have proven to be effective complementary methods of treatment for this problem.

The meta-analysis of Zeng et al. [25] included seven RCTs for a total of 689 subjects and showed a statistically significant reduction of fatigue at the end of treatment and of follow-up. However, owing to the heterogeneity and low methodological quality of the studies, the authors conclude that further research is necessary in order to confirm the effectiveness of acupuncture.

In their review and meta-analysis (2014) on the effect of moxibustion (indirect and with *Zingiber off.*) on cancer-related fatigue, Lee et al. [26] examined four Chinese-language RCTs (374 patients), which demonstrated a statistically significant effectiveness of the treatment with moxibustion added to the usual care compared to the usual care alone (*p* = 0.0003). The most used points were: CV4, CV6, CV8, CV12 and ST36. However, according to the authors, further higher quality studies are needed to draw conclusions on the effectiveness of moxibustion.

In 2014, Greenlee et al. (SIO) [27] published a document entitled “Clinical Practice Guidelines on the Use of Integrative Therapies as Supportive Care in Patients Treated for Breast Cancer”. This paper used a new classification system of effectiveness divided into six levels (A, B, C, D, H, I): acupuncture is assigned grade C (at least a moderate certainty that there is a small benefit) for pain, hot flashes, anxiety, depression, and fatigue. Acupressure and electroacupuncture (EA) for post-chemotherapy nausea and vomiting received grade B (high certainty that the net benefit is moderate or moderate certainty that the net benefit is moderate-to-substantial). However, more recent studies have evidenced a greater effectiveness of acupuncture (for pain and hot flashes).

### 2.1. Herbal Medicine

Medicinal herbs mitigate numerous symptoms related to conventional therapies used in cancer patients. These remedies already belong to the knowledge of traditional and popular medicine, so much so that the practices of self-medication, like the use of infusions to improve digestion, reduce nausea, anxiety, insomnia or constipation, are very wide-spread.

In more recent years, scientific research has increased considerably in terms of epidemiological investigations to assess chemo-preventive potentials, but in particular to assess the effectiveness of medicinal herbs on the control of symptoms related to oncological therapies, and the mechanism of pharmacological action. Alongside its traditional use, it is possible today to use herbal medicine in trials based on documented clinical evidence, generally to relieve the most common symptoms, when these prove to be drug resistant.

The most studied vegetal substances are *Panax ginseng* [28] graded 1A and, with less evidence, Guaranà (*Paullinia cupana*) graded 2B [29], both of which are used to fight fatigue; also highly studied is Aloe (*Aloe vera*) as treatment for mucositis related to anticancer therapies [30], graded1B. Very important is *Cannabis sativa* to control pain in cancer patients, graded 1A [31], and also side effects of opioids; and Ginger extracts (*Zingiber officinale*), graded 1A, to treat nausea and vomiting post-chemotherapy, for which Cannabis can also be used with a grade of 2B [32,33,34].

Lavender essential oil (*Lavandula officinalis*) [35] can be used orally and externally to alleviate anxiety (graded 1B), while Saffron (*Crocus sativus*) is useful in depressive syndromes [36], graded 1B, as well as Rhodiola (*Rhodiola rosea*) [37], graded 2C; the latter has already been registered as a “Traditional herbal medicinal product” in Europe.

These are examples of herbal remedies whose effectiveness has been documented in clinical trials also conducted on cancer patients. However, other medicinal plants can be used, since their pharmacological and clinical activities are known, even if they are tested and documented on non-cancer patients. For example, in our facilities we see many women in the post-surgery and post-chemotherapy stages of breast cancer experiencing muscle and joint pain caused by anti-estrogen therapy. In these cases, the remedies systematically used are herbs with anti-inflammatory activity, such as extracts of *Ribes nigrum* leaves and *Boswellia serrata* resin, with very positive results [38,39].

As also evidenced by extensive literature reviews, the preparations made from mistletoe (*Viscum album*) have proved to strengthen the defense capabilities of the affected body, although they rightfully belong to anthroposophic medicine. Though two systematic researches revealed a small number of qualitative studies of mistletoe therapy in cancer [40,41] specific extensive literature is also available on the use of mistletoe in breast, lung, pancreatic, and colorectal cancer which shows interesting positive results in terms of the reduction of related symptoms like pain, fatigue, loss of appetite, and insomnia, as well as an improvement of the quality of life [42,43,44,45,46].

It is worth remembering that, unlike acupuncture and homeopathy, which have very few side effects or interactions, the use of medicinal plants in oncology requires attention and specific high standard skills. Indeed, in modern phytotherapy, fundamental are the variability of the qualitative-quantitative composition of the plant; the patient’s individual response to therapy, linked to characteristics such as age, ethnicity, gender, weight, concomitant diseases etc.; the route of administration; the pharmacokinetic interferences interacting with absorption; metabolism; excretion of the substance; and the interferences on the pharmacodynamics of the substance [47].

In this respect, the antagonistic effects of some medicinal plants combined with common drugs (licorice and diuretics, vitamin K and anticoagulants, etc.) are known and, as is, conversely, the summation of the effects by other herbs. Representative of this is the case of Saint John’s wort (*Hypericum perforatum*), which has a good level of evidence of being an effective treatment of depression, but which is not used on cancer patients owing to its interference with anticancer protocols.

The increase in the use of herbal remedies in the form of uncontrolled self-medication also implies a greater exposure to the potential risks of drug interactions. There has been a growing number of reports concerning the interactions of medicinal herbs and anti-cancer drugs to the point that they are becoming a problem for patient safety. The induction and inhibition of the metabolic enzymes and membrane transporter systems are considered important mechanisms of interaction between herbs and anticancer drugs. Theoretically, the herb-drug interactions could be positive, negative, or neutral, and there is indirect evidence for both the positive and negative effects of the concomitant use of herbs and chemotherapeutic agents.

On the one hand, pre-clinical studies have demonstrated that a number of herbal medicines could increase the sensitivity of cancer cells to chemotherapy drugs, improve survival rates, enhance the tumor response to chemotherapy, and reduce the toxicity of chemotherapy, for example the case of *Curcuma longa* in colon cancer patients [48].

At the opposite end of the spectrum, other herbs like *Hypericum* have been reported to cause potential adverse pharmacokinetic interactions with anticancer drugs [49].

Some medicinal plants may, for example, increase the toxicity of certain drugs, as is the case of hepatitis from Imatinib in a patient who was also taking ginseng, known as an enzyme inhibitor of 3A4 cytochrome. Other herbs can instead reduce a drug’s effectiveness, such as green tea which behaves as an inductor of the same cytochrome, or which may reduce the clinical effectiveness of Bortezomib. Another example of this is grapefruit juice, which inhibits CYP 3A4, thus dangerously increasing the toxicity of chemotherapy and targeted therapies using this cytochrome. The knowledge and study of the interactions and of their mechanisms (including herbs and drugs) are very recent, and many are still unpredictable.

Taking these factors into account, the few pharmacological data available about the interactions with herbs offer a guide that is often hardly reliable for clinical practice and insufficient to provide definite recommendations.

In order to improve the clinical interpretation of the in vitro and/or in vivo interactions between medicinal plants and chemotherapy, the Tuscan Network of Integrative Medicine has suggested so-called “*reversed*” grading (see Table 1), where the principal level of evidence corresponds to the main level of the negative recommendation. This classification also allows the possibility to use and exploit the positive interactions (synergies) between medicinal plants and drugs.

### 2.2. Homeopathic Medicine

Positive studies have been published for homeopathy, mainly for the treatment of disorders related to suppressive hormonal therapy in women with hormone-dependent cancers, especially breast cancer.

According to these studies, homeopathy can be used to reduce the discomfort of vasomotor disturbances [50,51]. For this symptom, more recent researches have been conducted. For instance, a study was carried out between 2005 and 2011 in a single Italian center in two steps [52]; a phase II pilot study, during which ten patients received homeopathic treatment for three months (phase A), followed by a phase III randomized study, where 35 patients received either homeopathic treatment or a placebo for six months (phase B). Women with a history of breast cancer and without metastatic disease suffering from menopausal symptoms were included in the study. A five-point numerical scale was used to evaluate the severity of the menopausal symptoms. The sum of the scores (total score) at baseline and after three months (phase A) or six months (phase B) were compared. In the pilot study (phase A), a mean reduction in the total score of 2.27 (SD ± 0.59) and a statistically significant reduction of the severity of hot flashes (*p* = 0.01), vaginal dryness (*p* = 0.027) and headaches (*p* = 0.015) were observed. In the placebo-controlled study (phase B), a statistically significant difference in favor of the homeopathic treatment was reported for night sweats (*p* = 0.0097), gastric symptoms (*p* = 0.039) and the total score (*p* = 0.018).

The randomized, placebo-controlled, double-blind study carried out by Colau et al. in 2012 [53] considered 108 women ≥50 years of age, in menopause for <24 months and with ≥5 hot flashes per day treated with a registered homeopathic medicine containing *Actaea racemosa* (4CH), *Arnica montana* (4CH), *Glonoinum* (4CH), *Lachesis mutus* (5CH), and *Sanguinaria canadensis* (4CH), or identical placebo tablets. Oral treatment (2 to 4 tablets per day) was started on day 3 after study enrollment and was continued for 12 weeks. 101 women were included in the final analysis (intent-to-treat population: homeopathic treatment, *n* = 50; placebo, *n* = 51). The global hot flashes score (HFS) over the 12 weeks, assessed as the area under the curve (AUC) adjusted for baseline values, was significantly lower in the homeopathic group than in placebo group (mean ± SD 88.2 ± 6.5 versus 107.2 ± 6.4; *p* = 0.0411).

A randomized, placebo-controlled, double-blind, double-dummy, superiority, three-arm trial with 6-week follow-up was conducted by Macías Cortés et al. in 2015 [54] on 133 patients in peri- and post-menopause with moderate and severe depression (according to DSM-IV) treated with homeopathy and compared with Fluoxetine. After a 6-week treatment, the homeopathic group was more effective than the placebo group by five points in the Hamilton Scale. The response rate was 54.5% and the remission rate was 15.9%. No differences were observed among the groups in the Beck Depression Inventory, but the homeopathic group was superior to the placebo group in the Greene Climacteric Scale (8.6 points), while the Fluoxetine group was not different from the placebo group.

In 2016, a recent short paper by JL. Bagot [55] describes his experience of 15 years conducted on around 4000 patients who were administered about 6000 treatments using also homeopathic dilutions of chemotherapy drugs (hetero-isotherapy) to reduce the side effects of anti-cancer therapies and to improve their quality of life. A significant decrease in side effects, allergic reactions and late sequelae was observed in these patients.

In 2014, Gaertner et al. [56] presented a re-analysis of homeopathic patient data compared to control patient data from the Outpatient’s Unit “Homeopathy in Malignant Diseases” of the Medical University of Vienna. For patients suffering from advanced stages of cancer and surviving the first 6 or 12 months after diagnosis respectively, the results show that utilizing homeopathy gives a statistically significant (*p* < 0.001) advantage over control patients regarding survival time. Bearing in mind all limitations, the results of this retrospective study suggest that patients with advanced stages of cancer might benefit from additional homeopathic treatment until a survival time of up to 12 months after diagnosis.

In 2015, Frass et al. [57] presented the results of a pragmatic randomized controlled trial on 410 patients receiving standard anti-cancer therapy: 200 patients receiving only conventional treatment and 210 with individually selected homeopathic remedies in addition to regular conventional treatment. The quality of life (QoL) of these patients was measured with the questionnaire EORTC QLQ-C30 form version 3.0 as well as a specially developed form evaluating subjective feeling using visual analogue scales. This first prospective controlled study evaluating add-on classical homeopathy in cancer patients at the same center shows that the global health status plus the subjective well-being of cancer patients improved significantly (*p* < 0.0001) with homeopathy.

Homeopathy has also given positive results in the treatment of radiation dermatitis, as evidenced by the published papers [58,59,60], including a Cochrane Review [61], however patients’ cohorts are few in number in these studies, and they deserve to be continued with a larger number of participants.

Also interesting is the use of a homotoxicological mouthwash to improve chemotherapy-induced stomatitis [62]. In this regard, no relevant side effects or pharmacological interactions have been observed, making the use of homeopathic medicine particularly easy.

In pediatrics, all the cancer patients treated at the University Children’s Hospital in Bern between 2002 and 2011 [63] were retrospectively surveyed about their use of CAM. The data were collected from 133 patients with a response rate of 52%. Of these, 53% had used CAM (mostly classical homeopathy) and 25% of patients had received information about these therapies from the medical staff. The most frequent reason for choosing CAM was that parents thought it would improve the patients’ general condition. The most frequent reason for not using CAM was the lack of information; of those who used CAM, 87% perceived positive effects.

A double-blinded, randomized placebo-controlled trial (not yet published) recruited patients from three cancer centers in North India [64]. After surgery scheduled for post-operative radiotherapy, 160 patients were randomly categorized in either a homeopathy (*n* = 80) or a placebo group (*n* = 80).

The provider-assessed maximum grade of Common Terminology Criteria for Adverse Events (CTCAE) was the primary endpoint of the study. Secondary endpoints included Skindex-16, Skin Toxicity, Symptom Experience, and quality of life self-assessment. Assessment was performed at baseline, weekly throughout, and four weeks after radiotherapy. In total, 148 patients completed the trial (homeopathy, *n* = 76; placebo, *n* = 72). Follow-up showed significant differences in the maximum grade of radiation dermatitis by homeopathy. CTCAE toxicity was greater in the placebo group (*p* = 0.002). After treatment, the homeopathy group showed less itching (*p* < 0.0001), less irritation (*p* < 0.0001), less symptom persistence or recurrence (*p* = 0.000), less annoyance with skin problems (*p* = 0.002), and less burning sensation (*p* = 0.002). Moreover, during the follow-up period, fewer homeopathy patients (23.6%) developed dermatitis compared to the placebo group (77.8%), indicating a more rapid improvement of these patients.

On the basis of literature evidence, the grading assigned to homeopathic treatment according to general criteria established by the SIO is the following: hot flashes, radiodermatitis, and pain (post-traumatic, including surgery), 1B; diarrhea, cancer-related fatigue, mucositis, anxiety and depression, 1C; insomnia, neuropathy, xerostomia, and oedema 2C.

To date, no sufficient evidence exists on the treatment of tumor progression. The studies are numerically limited and not always of good methodological quality. Research is also trying to identify active principles that can interfere with the progression of the tumor as chemo-preventive, pro-apoptopic, immunostimulant, cytostatic agents, and as inhibitors of the replication of cancer cells.

## 3. Integrative Oncology in the Public Health Systems: The Experience of the Region of Tuscany

The experiences of using CM in oncology are differently modulated in the clinical setting, but the most common approach is that of integrative oncology, defined as “both a science and a philosophy that focuses on the complexity of the health of cancer patients and proposes a multitude of approaches to accompany the conventional therapies of surgery, chemotherapy, molecular therapeutics, and radiotherapy to facilitate health” [65].

The integration of CM in oncology, in particular within Comprehensive Cancer Care Networks (CCCN), should first of all take place in accordance with the principles of relevance to cancer patient assistance. Therefore, it is fundamental that the principles and methodology of the interventions of complementary medicine are comprehensible to patients, healthcare professionals and conventional oncologists, and that they are economically sustainable.

Another step consists in defining therapeutic protocols for the treatment of the symptoms of the disease and of the side effects of cancer therapies for each complementary medicine and for the models of integrative care; in particular, in defining how the different therapies should be associated in a synergistic way in order to improve the effectiveness of cancer treatments. Utmost attention must be paid to the issue of patient safety and to the management of the specific clinical risk, by identifying criteria for the safety of the structures, of the specific methods of each discipline, with particular attention paid to the risk of pharmacological interactions.

Clinical outcome will be used to assess the impact (in terms of effectiveness/efficiency) of complementary interventions; questionnaires concerning the approval and evaluation of the quality of life of the patients; impact on the structure and on the network; and long-term evaluations on survival.

Last but not least, the entire integrative approach requires adequate training of the personnel, to be implemented with processes of basic and continuous education, with particular attention paid to the correct dissemination of the results to the public and to health professionals engaged in the process.

The Region of Tuscany has developed a process of integration of CM in its Regional Health Service over a number of years. This has allowed the inclusion of acupuncture, herbal medicine, homeopathy and manual medicine in the Essential Levels of Healthcare of the region; the setting up of about one hundred public hospitals or clinics providing an approximate 35,000 annual treatments; the establishment of the Tuscan Network of Integrative Medicine, the clinical government structure at the Department of Regional Healthcare, and four regional Referral Structures for TCM/acupuncture, herbal medicine, homeopathy and integrated medicine in a hospital setting.

This integration in Health Public Service is an element of a strong guarantee for the health of citizens, since CM are subject to the same rules of official medicine and must respond to the criteria of quality and appropriateness of the healthcare services.

The integration of therapies is the most reasonable and correct choice, to avoid cancer patients using “unofficial” treatment as a form of self-medication, regardless of scientific checks and necessary quality and safety requirements, and exposing themselves to the risk of possible drug interactions or of reduced compliance with anticancer official protocols.

Within this framework, the need has emerged to plan an “evidence-based” intervention for integrative cancer treatment and to promote research in order to clarify whether complementary therapies have an impact on the survival of cancer patients and help improve their well-being and quality of life.

On the basis of literature reviews (the major steps of which have been summarized in the previous paragraphs), and by means of a constant dialogue with the Tuscan Tumor Institute (TTI), the Tuscan Regional Council Resolution 418 in 2015 defined the modes of integration of complementary treatments in the regional oncology network.

The treatments that have been assigned 1A (strong recommendation, high quality evidence) or 1B (strong recommendation, moderate-quality evidence) grading will be fully integrated into health services for cancer patients.

For acupuncture and TCM, the areas of application are post-chemotherapy and post-surgical nausea and vomiting, pain in all of its phases, vasomotor disturbances of iatrogenic menopause and xerostomia; for homeopathy, the vasomotor disturbances of iatrogenic menopause and adverse effects of radiotherapy; for herbal medicine, cancer-related fatigue, nausea and vomiting, pain, mucositis, anxiety and depression. In parallel, clinical research projects will be started to assess effectiveness, side effects and interactions with the official treatment of CM treatments that have collected less evidence.

On the basis of documented evidence, complementary medicine treatments made available to Tuscan citizens have shown to be safe and effective with this measure.

These therapies will be integrated in the Tuscan Network of Oncology Departments, operating in synergy with public hospitals or clinics of CM and in coordination with the Regional referral structures for complementary medicine.

The already active Tuscan public hospitals and clinics of integrative oncology are the following:

- Centre “Fior di Prugna” (Regional Referral Structure for TCM and CM), Local Health Unit Tuscany Center, Florence
- Breast Unit, Local Health Unit Tuscany Center, Florence
- Clinic “Complementary Medicine and diet in oncology”, Clinic of homeopathy (Regional Referral Structure for homeopathy) Local Health Unit, North West Lucca
- Clinic of phytotherapy—CERFIT of the University Hospital of Careggi, Regional Referral Structure for phytotherapy
- Department of Oncology—SOD Anesthesia and Intensive Therapy of the University Hospital of Careggi, Florence
- Complementary Medicine and integrative oncology, Breast Unit of the University Hospital of Pisa
- Centre of integrated medicine of the Hospital of Pitigliano and Unit of Palliative Treatment, Local Health Unit Tuscany South East, Grosseto
- Hospital of Empoli, Local Health Unit Tuscany Center
- Hospital of Prato, Local Health Unit Tuscany Center

## 4. Discussion

Cancer is a systemic and multifactorial disease and, as such, can benefit from the synergistic use of multiple therapies. The multidisciplinary approach is the route to be pursued, with the aim of always selecting the best therapy for each patient and of implementing real “Comprehensive Cancer Care.”

The literature evidences that additive CM and, specifically, acupuncture and TCM, herbal medicine and homeopathy can be applied effectively to cancer patients in the clinical situations described. This research development, which remains a work in progress, has increased the possibility of creating an integrated therapeutic approach for cancer patients who, like all citizens, have the right to use evidence-based and safe complementary therapies.

In the following years, new studies on larger samples and with strict criteria must be conducted to develop and consolidate the effectiveness of the evidence so far available; special attention must be paid to reducing the side effects of anti-cancer treatments (chemotherapy, radiotherapy, surgery, hormone therapy), and to the quality of life of cancer patients. Moreover, utmost attention should always be paid to the issue of the patient’s safety and to the management of the specific clinical risk, with particular attention paid to pharmacological interactions.

In this path towards integration, the exchange of knowledge and experiences is fundamental and, therefore, it is necessary for physicians (primarily the oncologists), and healthcare professionals involved in the cancer field to be appropriately informed about the potential benefits of complementary medicines.

These procedures and the process as a whole define the integration of complementary treatment and drugs of proven effectiveness within a Global Cancer Network by ensuring the inclusion of a “new therapeutic offer” in cancer networks.

Finally, they make it possible to respond safely and effectively to many cancer patient demands, when these therapies are evidence-based and therefore able to harmonize equity of access and patient safety.

## 5. Conclusions

As shown by the literature, the integration of evidence-based complementary treatments effectively responds to the demand coming from cancer patients and combines safety and equity of access in public health systems.

In this regard, it is essential to define therapeutic protocols for the treatment of the symptoms of the disease and of the side effects of cancer therapies for each complementary medicine and for the models of integrative care. CM are usually considered personalized therapies, nevertheless it is possible to draw up some protocols in order to reduce the adverse effects of conventional drugs which cause the same symptoms on the patient. In some cases, different therapies, such as homeopathy, acupuncture or herbal medicine, can be synergistically associated to improve the effectiveness of anti-cancer treatments. In other cases, the same therapies can be used in succession by evaluating the patient’s response to the single therapy each time.

The integration of CM in oncology should take place in accordance with the principles of relevance to cancer patient assistance. Therefore, it is fundamental that the principles and methodology of the interventions of CM are comprehensible to patients, healthcare professionals and conventional oncologists, and that they are economically sustainable.

## Figures and Tables

**Table 1:** Reversed Grading. I–V grade of evidence correlated to risk. A (Abstain), B (Prescription).

Reversed Grading	Evidence	Recommendation
IA	Laboratory evidence in vitro and in vivo and clinical reports of pharmacological interference with proven risk of effectiveness reduction of anticancer therapy	ABSTAIN from prescription during oncological therapy
IIA	Only in vitro and in vivo laboratory evidence without any signalling of clinic interference, and no research carried out to study the clinical interactions in oncology	Evaluate whether to ABSTAIN or NOT from prescription on the basis of a risk/benefit assessment of therapy. Stop the administration in the presence of reduced effectiveness or ineffectiveness of anti-cancer therapy and/or adverse effects
IIB	Only in vitro and in vivo laboratory evidence, without any signalling of clinic interference in spite of the research carried out	PRESCRIPTION and monitoring. Stop the administration in the presence of reduced effectiveness or ineffectiveness of anti-cancer therapy and/or adverse effects
IIIB	Only in vitro laboratory evidence (NOT in vivo), without any signalling of clinic interference in spite of the research carried out	PRESCRIPTION and monitoring. Stop the administration in the presence of reduced effectiveness or ineffectiveness of anti-cancer therapy and/or adverse effects
IVB	No evidence of negative interference, rather, positive evidence of oncological-therapy potentiation	PRESCRIPTION and monitoring. Report to the reference oncologist for any reformulation in the dose of anticancer treatment and/or adverse effects
VB	No evidence of negative interference and positive evidence of oncological therapy potentiation	PRESCRIPTION. Report to the reference oncologist
